# Monitoring mitochondrial inner membrane potential for detecting early changes in viability of bacterium-infected human bone marrow-derived mesenchymal stem cells

**DOI:** 10.1186/scrt144

**Published:** 2012-12-11

**Authors:** Mika Pietilä, Kaarina Lähteenmäki, Siri Lehtonen, Hannu-Ville Leskelä, Marko Närhi, Maarit Lönnroth, Jaana Mättö, Petri Lehenkari, Katrina Nordström

**Affiliations:** 1 Institute of Biomedicine, Department of Anatomy and Cell Biology, Aapistie 7, P.O. Box 5000, FIN-90014, University of Oulu, Oulu, Finland; 2 Institute of Clinical Medicine, Division of Surgery, University of Oulu and Clinical Research Centre, Department of Surgery and Intensive Care, Aapistie 5a, P.O. Box 5000, FIN-90014, Oulu University Hospital, Oulu, Finland; 3 Finnish Red Cross Blood Service, Kivihaantie 7, FI-00310, Helsinki, Finland; 4 Aalto School of Chemical Technology, Department of Biotechnology and Chemical Technology, Kemistintie 1A, P.O. Box 6100, 00076, Aalto, Finland

## Abstract

**Introduction:**

One of the most challenging safety issues in the manufacture of cell based medicinal products is the control of microbial risk as cell-based products cannot undergo terminal sterilization. Accordingly, sensitive and reliable methods for detection of microbial contamination are called for. As mitochondrial function has been shown to correlate with the viability and functionality of human mesenchymal stem cells (hMSCs) we have studied the use of a mitochondrial inner membrane potential sensitive dye for detecting changes in the function of mitochondria following infection by bacteria.

**Methods:**

The effect of bacterial contamination on the viability of bone marrow-derived mesenchymal stem cells (BMMSCs) was studied. BMMSC lines were infected with three different bacterial species, namely two strains of *Pseudomonas aeruginosa*, three strains of *Staphylococcus aureus*, and three strains of *Staphylococcus epidermidis*. The changes in viability of the BMMSCs after bacterial infection were studied by staining with Trypan blue, by morphological analysis and by monitoring of the mitochondrial inner membrane potential.

**Results:**

Microscopy and viability assessment by Trypan blue staining showed that even the lowest bacterial inocula caused total dissipation of BMMSCs within 24 hours of infection, similar to the effects seen with bacterial loads which were several magnitudes higher. The first significant signs of damage induced by the pathogens became evident after 6 hours of infection. Early changes in mitochondrial inner membrane potential of BMMSCs were evident after 4 hours of infection even though no visible changes in viability of the BMMSCs could be seen.

**Conclusions:**

Even low levels of bacterial contamination can cause a significant change in the viability of BMMSCs. Moreover, monitoring the depolarization of the mitochondrial inner membrane potential may provide a rapid tool for early detection of cellular damage induced by microbial infection. Accordingly, mitochondrial analyses offer sensitive tools for quality control and monitoring of safety and efficacy of cellular therapy products.

## Introduction

Stem cell therapies have raised hopes for the development of viable alternatives for the treatment of many severe degenerative and inflammatory conditions that currently cannot be cured or alleviated by traditional medicine [1-5]. However, there are still many important issues that need to be solved before the transfer of these advanced cellular therapies from the laboratory to clinics can be successfully achieved. One of the major challenges of such products and therapies is ensuring product safety while maintaining efficacy since viable stem cell-based products cannot undergo terminal sterility prior to implantation and use [6]. Accordingly, the development of aseptic manufacturing processes and process controls is pivotal to the successful translation of research into clinical practice [7]. In addition, the short shelf-life of products calls for the development of rapid methods to detect bacterial contamination in a matter of hours [8,9].

Even though the incidents of microbial contamination of cell products seem to be rare, the risk of contamination is a critical issue since the consequences to patient health may be unpredictable and even devastating [10-16]. Currently, there is little or no evidence to predict the long-term outcomes of possible infections after cell implantation. Therefore, uncertainties of possible further transmission of infectious agents from cell or tissue implants to patient exist. Moreover, microbial damage to stem cell products prior to implantation may well lead to unpredictable consequences in the efficacy of the implanted cells. Accordingly, strict requirements set by regulatory agencies for the manufacture of cell-based products, which must be performed according to the principles of good manufacturing practices or good tissue practices [17]. Even so, the most common site for contamination is the *in vitro *expansion and manipulation of cells [14,15], and the most frequent sources of contamination are human skin, the environment, clothing, and gowning of hospital staff [12,15,18,19]. A variety of different pathogens have been isolated from cellular products, and the most common is coagulase-negative *Staphylococcus spp*. [10-16]. Moreover, a recent study has elucidated the ability of different pathogens, namely *S. aureus*, *S. epidermidis*, and *Pseudomonas aeruginosa*, to adhere to a biomaterial surface in the presence and absence of macrophages [20]. Despite the presence of macrophages, osteoblast-like cells lost the race of growth area on the biomaterial surface in the presence of *S. aureus *or *P. aeruginosa*, but the cells survived about 48 hours in the presence of *S. epidermidis*. These findings highlight the importance of microbial risk management in the prevention of biomaterial-associated infections.

Regulatory demands for the manufacture of stem cell-based medicinal products in the European Union dictate certain requirements for their quality, safety, and efficacy [21]. However, even though there are a number of established methods for the control of conventional biological medicinal products, most of them are poorly applicable to the control of the manufacturing processes of cell-based products. Moreover, methods that can be used are often laborious and time-consuming [22-25]. Therefore, one possible avenue to be explored in microbial risk management is the development of methods for monitoring of the mitochondrial status of the cells in the intended product. Mitochondria are the powerhouses of cell and are responsible for the main energy production and are also an important regulator of apoptosis [26-28]. In addition, it has recently been shown that, in stem cell biology, mitochondria have a special role in the regulation of the ultimate fate of stem cells [29-33]. Accordingly, we have focused on mitochondrial analysis as a tool for measuring the efficacy and potency of stem cell-based cellular products intended for clinical applications [33]. Recently, we have shown that by analyzing mitochondrial inner membrane potential (ΔΨ_m_) it is possible to detect early changes in viability and also to predict the osteogenic differentiation potency of human mesenchymal stem cells (hMSCs). We therefore postulate that monitoring of ΔΨ_m _could also be used as a tool to detect early changes in viability of infected hMSCs and thus monitor the microbiological safety of stem cell products.

In the present study, the viability of human bone marrow-derived human mesenchymal stem cells (BMMSCs) was studied after deliberate and controlled cell culture infection in co-culture with eight different bacterial strains. The bacteria included two strains of *P. aeruginosa*, three strains of *Staphylococcus aureus*, and three strains of *Staphylococcus epidermidis*. *P. aeruginosa*, which is a Gram-negative rod, and the Gram-positive cocci *S. aureus *and *S. epidermidis *have been shown to be common contaminants of cell products and in the hospital environment [10-16,34]. Two of the bacterial strains had previously been isolated from discarded, contaminated batches of blood products, making them biologically relevant models for studying the contamination process of stem cell cultures. The effects of the bacterial strains on ΔΨ_m_, cell morphology, and plasma membrane integrity of BMMSCs were studied.

## Materials and methods

### BMMSC isolation and culture

BMMSC lines 407, 412, and 470 were isolated from unaffected bone sites of patients who underwent surgery for osteoarthritis at Oulu University Hospital in Finland. These cell lines have been approved for research use by permission of the Northern Ostrobothnia Hospital District Ethical Committee. Samples from bone marrow were suspended in a proliferation medium containing alpha minimum essential medium (αMEM) (Sigma-Aldrich, St. Louis, MO, USA) buffered with 20 mM HEPES and containing 10% heat-inactivated fetal bovine serum (FBS), 100 U/mL penicillin, 0.1 mg/mL streptomycin, and 2 mM L-glutamine. The suspension was transferred into cell culture flasks, and BMMSCs were allowed to attach for 48 hours at 37°C under 5% CO_2 _and 20% O_2_. Nonattached cells were removed by changing fresh medium, and attached cells were cultured at the bottom of the flask until they reached a 70% to 80% confluence. The medium was changed twice a week, and cells from passages 3 to 5 were used in experiments.

### Flow cytometric analysis of surface antigens

The BMMSCs were characterized according to the minimal criteria panel of surface antigens proposed by the Mesenchymal and Tissue Stem Cell Committee of the International Society for Cellular Therapy [35]. BMMSC lines 407, 412, and 470 were detached from culture flasks and suspended in phosphate-buffered saline (PBS) + 0.5% bovine serum albumin (BSA) (100,000 cells/mL). Conjugated antibodies were incubated for 20 minutes at room temperature: CD90 (FITC; StemCell Technologies, Vancouver, BC, Canada), CD73 (PE; BD Biosciences, San Jose, CA, USA), HLA-ABC (APC; BD Biosciences), and CD105 (FITC; Abcam, Cambridge, UK). Negative surface antigens for hMSCs were incubated simultaneously as a group in the same sample: HLA-DR (PE; BD Biosciences), CD34 (PE; BD Biosciences), CD45 (PE; BD Biosciences), CD14 (PE; BD Biosciences), and CD19 (PE; BD Biosciences). After incubation, cells were washed with PBS + 0.5% BSA and analyzed by using a FACSCalibur instrument (Becton, Dickinson and Company, Franklin Lakes, NJ, USA), equipped with a laser emitting at 488, 633, and 407 nm. FITC, PE, and APC channels were used to detect the emission of conjugated surface antigens. Flow cytometric data were analyzed with FlowJo. Cell debris was gated out from all samples. On the basis of isotype controls and unlabeled cells, the gates for positive cells were defined.

### Bacterial strains and cultivation condition

*P. aeruginosa *PA01 (VTT E-84219), *S. aureus *Cowan I (DSM 20372), and *S. epidermidis *ATCC 14990 were obtained from commercial culture collections. *S. aureus *KK1089 and *S. epidermidis *KK1087 were isolated from discarded, contaminated batches of thrombocyte preparations. The bacterial strains were stored in skim milk suspension at −80°C. Before co-culture experiments, bacteria were plated onto trypticase soy agar (TSA) plates (Tammer Tutkan Maljat Ltd., Tampere, Finland) and cultivated at 37°C overnight. Following incubation, a single colony was inoculated into 5 mL of soy casein broth (Tammer Tutkan Maljat Ltd. Tampere). The bacterial suspensions were cultivated at 37°C overnight. The next morning, bacteria were pelleted by centrifuging at 2,465*g *for 10 minutes, washed twice with 10 mL of PBS, pH 7.2, and finally suspended in 1 mL of PBS. The bacterial suspensions were adjusted to an optical density of 1.0 at A_600 _nm by Shimadzu UV-1700 Pharma Spec spectrophotometer (Shimadzu Corporation, Kyoto, Japan). Based on previous experience, this corresponds to an order of magnitude of 10^8 ^bacteria/mL. Viable counting of bacterial suspensions in different experiments showed that bacterial numbers ranged from 9 × 10^7 ^to 3 × 10^8 ^CFU/mL for *S. aureus *strains, from 1 × 10^8 ^to 5 × 10^8 ^CFU/mL for *S. epidermidis *strains, and from 4 × 10^8 ^to 7 × 10^8 ^CFU/mL for *P. aeruginosa *PA01. Series of 10-fold dilutions of all bacterial suspensions were performed in PBS, and three dilutions of each bacterial strain were used to infect BMMSCs. Small bacterial inocula (approximately 10^1 ^to 10^3 ^CFU) were used in order to simulate the aseptic cell production process, in which contaminations are most likely to derive from infection with only a few bacterial cells, followed by bacterial multiplication in the nutrient-rich cell culture medium. Moreover, previous studies on the interaction of bacteria with MSCs or osteoblast-like cells have similarly used bacterial amounts ranging from 3 × 10^2 ^to 1 × 10^3 ^[20,36].

*P. aeruginosa *ATCC9027, *S. aureus *ATCC6538, and *S. epidermidis *ATCC12228, which are available as certified reference material, were obtained from Microbiologics (Labema OY, Kerava, Finland as lyophilized preparations that contain a specified number of bacteria. The preparations were dissolved in accordance with the instructions of the manufacturer. The number of recovered bacteria was verified by viable counting. The final amounts of bacteria used were 2 × 10^2 ^CFU/mL for *P. aeruginosa *ATCC9027, 4 × 10^2 ^CFU/mL for *S. aureus *ATCC6538, and 3 × 10^2 ^CFU/mL for *S. epidermidis *ATCC12228.

### Bacterial infection of BMMSCs

BMMSCs were first cultivated as described above with antibiotics and then were washed with PBS, trypsinized from culture flasks, and suspended in the expansion medium without antibiotics. BMMSCs were plated on clear 96-well plates for Trypan blue staining and microscope analysis and in parallel on black (clear bottom) 96-well plates for ΔΨ_m _analysis. BMMSCs were plated as 10,000 cells per well and cultured overnight at 37°C under 5% CO_2 _and 20% O_2_. Bacterial dilutions corresponding approximately to 10^1^, 10^2^, or 10^3 ^bacteria per well were inoculated, each individual strain separately, into the 96-well plates in which BMMSCs had been grown overnight. For the ΔΨ_m _analysis, carbonyl cyanide m-chlorophenylhydrazone (CCCP) (Sigma-Aldrich; 10 μM) was used as a positive control. All treatments were performed as four independent replicates. The ΔΨ_m _analysis was performed after 4 hours of infection and Trypan blue staining, and observation of cellular morphology was performed after 3, 4, 6, and 24 hours of infection.

### Determination of bacterial growth

Bacterial growth was determined after 4 and 24 hours of infection of the BMMSC line 407. As a control, bacterial growth was determined also after 4- and 24-hour incubation under identical culture conditions and in a medium not containing BMMSCs. Series of 10-fold dilutions of bacterial suspensions were performed in PBS from all of the analyzed samples, and bacteria from different dilutions were plated onto TSA plates and cultivated at 37°C overnight. The next morning, colonies were calculated and the number of CFU was determined. Bacterial loads after incubation were compared with bacterial amounts in the inocula.

### Analysis of mitochondrial inner membrane potential

The infection of BMMSCs was terminated after 4 hours by removing the bacterium-containing medium and washing two times with PBS. After washing, the mitochondrial inner membrane potential sensitive dye enhanced 5,5',6,6'-tetrachloro-1,1',3,3'-tetraethylbenzimidazolcarbocyanine iodide (JC-10) (Enzo Life Sciences, New York, NY, USA) was used. The JC-10 staining solution (5 μM) was prepared by diluting the JC-10 stock solution (1.7 mM in DMSO) into the expansion medium. The staining solution was mixed well, added to the cells, and incubated for 60 minutes at room temperature. Following incubation, the staining solution was removed and PBS was added. Expansion medium in wells without cells was used as a background, and JC-10 staining was performed as for the wells with BMMSCs. The JC-10 signal was detected by a fluorescence plate reader (Victor^2 ^1420 Multilabel counter; Wallac, now part of PerkinElmer Inc., Waltham, MA, USA). Fluorescence was excited at 488 nm, and the emission was measured at 595 ± 42 nm and 535 ± 30 nm. The background fluorescences of 595 and 535 nm from wells without cells were subtracted from the analyzed samples. The 595 nm/535 nm emission ratio was determined from four different replicates, and the ratio of infected or CCCP-treated cells was compared with the control cells. CCCP was used to depolarize ΔΨ_m_, which was seen as a decrease in the 595 nm/530 nm emission ratio.

### Trypan blue staining

Trypan blue (Stem Cell Technologies, Vancouver, BC, Canada) was diluted 1:100 in PBS and added onto the cells. Trypan blue staining, together with photography, was performed after 3, 4, 6, and 24 hours of infection. Cells were studied by using an inverted microscope Leica DM IL LED (Leica, Wetzlar, Germany) and photographed with a Leica DFC 420 digital microscope camera.

### Statistics

Statistical analysis and all diagrams were performed by using the OriginPro version 8 (OriginLab Corporation, Northampton, MA, USA). JC-10 data are presented as mean ± standard deviation (SD) of the results from four independent replicates. The significance level was determined by two-sample *t *test. A *P *value of less than 0.05 was considered statistically significant.

## Results

### Even low numbers of pathogens damage BMMSCs within 24 hours

**Figure 1:**
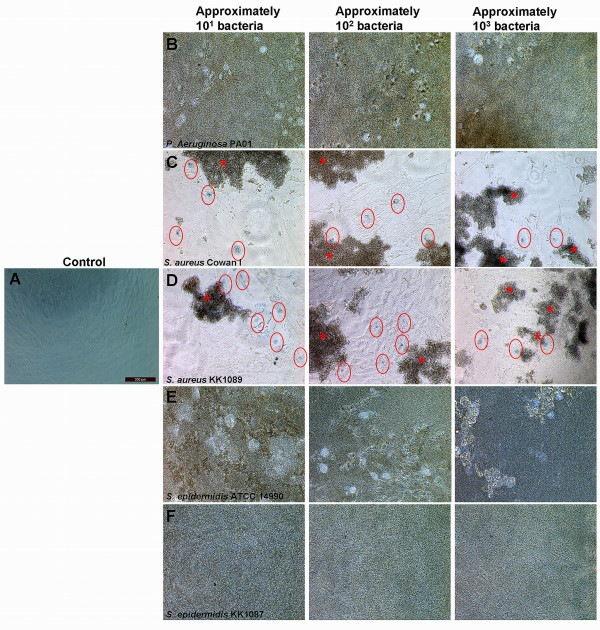
**Total dissipation of human bone marrow-derived mesenchymal stem cells (BMMSCs) by bacterial infection**. BMMSCs (line 412) were infected with bacteria, and after 24-hour incubation the cells were stained with Trypan blue and studied microscopically. **(a) **Control cells were viable after 24 hours of cultivation. **(b) ***Pseudomonas aeruginosa *PA01-infected BMMSCs show total dissipation at 24 hours with all bacteria loads. **(c) ***Staphylococcus aureus *Cowan I and **(d) ***S. aureus *KK1089 formed enormous aggregates and caused the decrease in viability as determined by Trypan blue. **(e) ***Staphylococcus epidermidis *ATCC 14990 and **(f) ***S. epidermidis *KK1087 showed total dissipation of BMMSCs at 24 hours of infection. Magnification was 200 × in all panels. Red circles indicate Trypan blue-positive nuclei. Red asterisks indicate the aggregates produced by *S. aureus*.

Bacterial infection of BMMSCs was studied in three BMMSC lines, namely 407 (female donor, 57 years old), 412 (male donor, 51 years old), and 470 (female donor, 70 years old). All of the BMMSC lines expressed cell surface antigens characteristic for hMSCs as proposed by the Mesenchymal and Tissue Stem Cell Committee of the International Society for Cellular Therapy [35] (Table 1). Bacteria were first incubated in three different amounts with BMMSCs (line 412). To determine the period of time that the bacterial contamination needs to induce total dissipation of BMMSCs even with the lowest bacterial load, we first decided to monitor the infection of BMMSCs for 24 hours. The effect of infection was compared with the uninfected control of BMMSCs (Figure 1a). The results show that *P. aeruginosa *PA01 caused total destruction of BMMSCs within 24 hours of infection (Figure 1b). *S. aureus *Cowan I and *S. aureus *KK1089 produced enormous aggregates, and cell death of BMMSCs was evident due to the appearance of Trypan blue-positive nuclei (Figure 1c, d). *S. epidermidis *ATCC 1499 and *S. epidermidis *KK1087 also caused total dissipation of BMMSCs after 24 hours of infection (Figure 1e, f). All pathogens caused a similar destruction of BMMSCs regardless of the initial amount of inoculated bacteria. This experiment was repeated with the BMMSC lines 407 and 470 and the results were consistent (data not shown).

**Table 1:** Detailed characterization of human mesenchymal stem cell lines used in this work

hMSC line	Positivity, percentage					Age in years/Gender
		
	CD90	CD105	CD73	HLA-ABC	CD14, CD19, CD34, CD45, HLA-DR	
407	98.2	99.8	100	99.5	1.7	57/Female

470	99.8	99.5	99.6	98.8	2.1	70/Female

412	100	99.6	99.9	99.4	1.1	51/Male

The positivity for the particular cell surface antigen was determined according to the negative control by flow cytometry. The panel of negative cell surface antigens was analyzed as a group. hMSC, human mesenchymal stem cell.

### Decrease in viability of BMMSCs after 6 hours of infection

**Figure 2:**
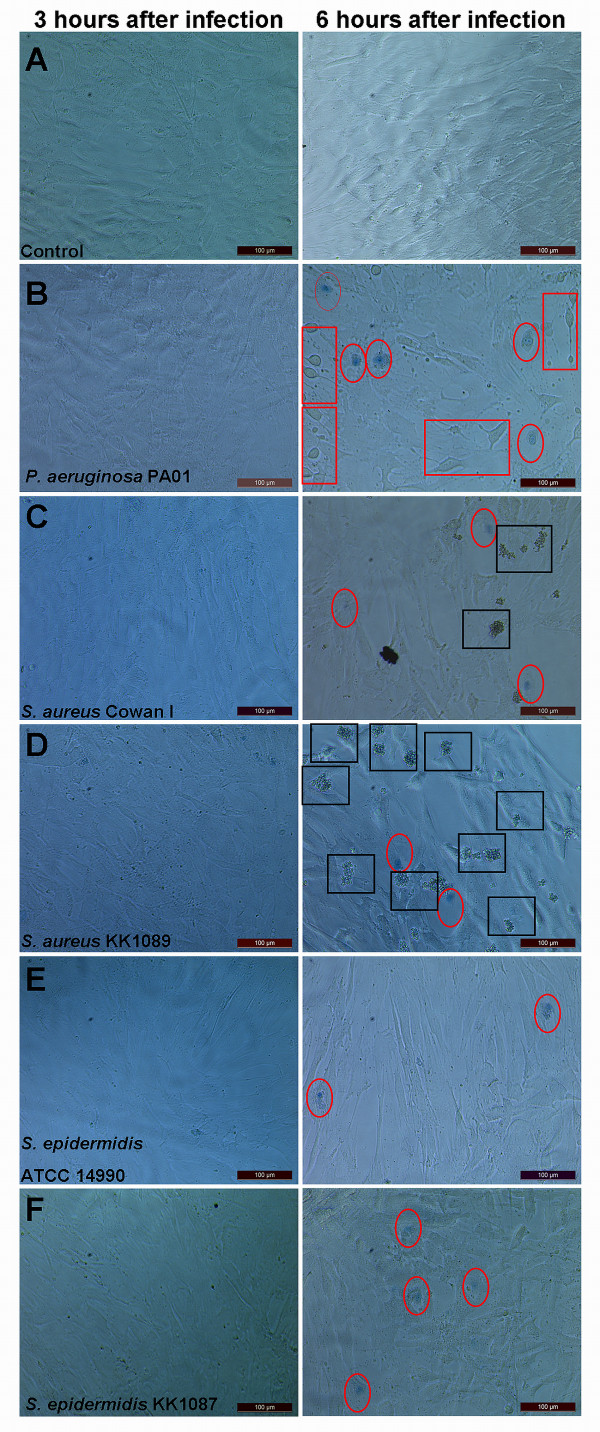
**Morphological changes induced in bone marrow-derived mesenchymal stem cells (BMMSCs) by bacterial infection**. BMMSCs (line 412) were infected with bacteria, and after 3- and 6-hour incubation the cells were stained with Trypan blue and studied microscopically. An inoculum size of approximately 10^3 ^bacteria was selected for experiments. **(a) **Control cells were Trypan blue-negative after 3 and 6 hours of culture. **(b) ***Pseudomonas aeruginosa *PA01-infected cells at 3 and 6 hours. After 6 hours of infection, the morphological changes were seen as a rounding of the cells and thus they lost their characteristic spindle-like morphology. **(c) ***Staphylococcus aureus *Cowan I- and **(d) ***S. aureus *KK1089-infected cells at 3 and 6 hours. Both *S. aureus *strains formed aggregates after 6 hours of infection. **(e) ***Staphylococcus epidermidis *ATCC 14990- and **(f) ***S. epidermidis *KK1087-infected cells at 3 and 6 hours. The morphology of the cells remained normal, but some Trypan blue-positive nuclei were evident. Magnification was 200 × in all panels. Red circles indicate Trypan blue-positive nuclei. Red squares indicate changes in morphology. Black squares indicate the *S. aureus*-produced aggregates.

The early effects of bacterial contamination on the viability of BMMSCs were studied by inoculating cells with approximately 10^3 ^bacteria per well and monitoring the cellular changes during the first 6 hours. Inoculated pathogens appeared to spend 3 hours in a lag phase most probably adapting to new culture conditions as during that time there were no differences between control cells (Figure 2a) and bacterium-infected cells (Figure 2b-f, photographs on the left column). After 6 hours of infection, however, changes in BMMSC viability became evident when compared with uninfected control cells (Figure 2a, photograph on the right column). *P. aeruginosa *PA01 caused a clear decrease in viability as determined by Trypan blue staining, and morphological changes in the BMMSCs were evident (Figure 2b, photograph on the right column). The morphological changes were seen as a rounding of the cells and loss of the characteristic spindle-like cell morphology. *S. aureus *Cowan I and *S. aureus *KK1089 promoted formation of small aggregates after 6 hours of infection, and some Trypan blue-positive nuclei were evident, whereas the morphology of BMMSCs appeared to stay normal (Figure 2c, d, photographs on right). *S. aureus *KK1089 showed the formation of aggregates in experiments in which lower numbers of bacteria (10^1 ^and 10^2^) were initially seeded, but such aggregates were not evident with *S. aureus *Cowan I (data not shown). Similar kinds of aggregates were also evident when *S. aureus *was grown in the medium without BMMSCs (data not shown). The infection of BMMSCs with *S. epidermidis *ATCC 14990 and *S. epidermidis *KK1087 showed some Trypan blue-positive staining of nuclei, but no clear morphological changes were evident in BMMSCs when compared with the uninfected control BMMSCs (Figure 2e, f, photographs on the right column).

### Pathogen-induced depolarization of ΔΨ_m _precedes major changes in cell morphology or decreases in viability of BMMSCs

**Figure 3:**
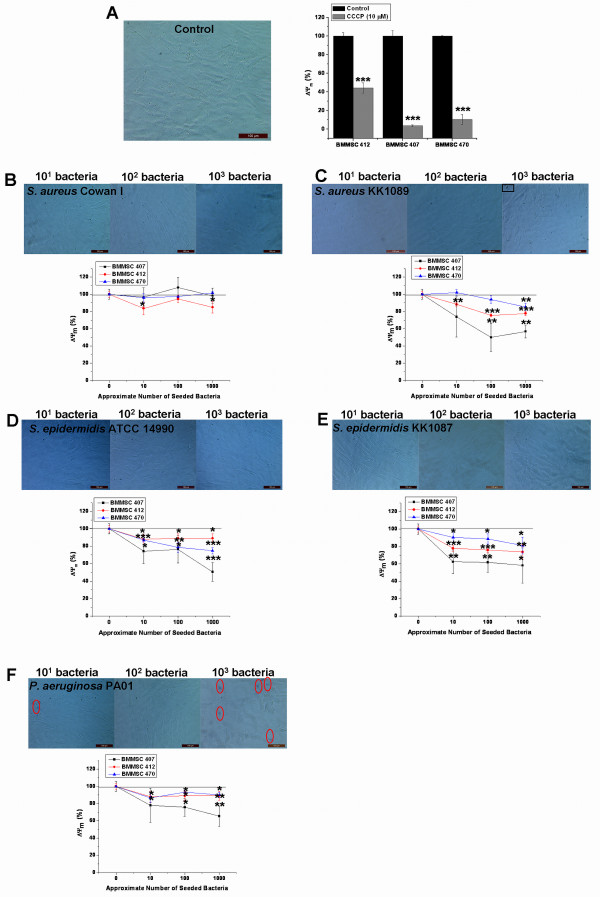
**Depolarization of ΔΨ_m _precedes changes in morphology or plasma membrane integrity of bone marrow-derived mesenchymal stem cells (BMMSCs)**. BMMSC lines 412, 407, and 470 were used to study the early changes in ΔΨ_m _and cell viability as determined by Trypan blue after 4 hours of infection. **(a) **Trypan blue staining of control cells and response to CCCP (protonophore) that was used as a positive control. **(b) ***Staphylococcus aureus *Cowan I caused slight depolarization of ΔΨ_m_. **(c) ***S. aureus *KK1089 induced changes in ΔΨ_m _and Trypan blue after 4 hours. **(d) ***Staphylococcus epidermidis *ATCC 14990 and **(e) ***S. epidermidis *KK1087 induced depolarization of ΔΨ_m_. **(f) **Depolarization of ΔΨ_m _and Trypan blue staining at 4 hours in *Pseudomonas aeruginosa *PA01-infected BMMSCs. The fluorescent 595 nm/535 nm ratio of JC-10 was used to determine ΔΨ_m_, and the 595 nm/535 nm ratios of infected BMMSCs were compared with uninfected control cells. Results are indicated as mean ± standard deviation of four independent replicates. Magnification was 200 × in all panels. Red circles indicate Trypan blue-positive nuclei. Black squares indicate the aggregates produced by *S. aureus*. *P <0.05, **P <0.01, and ***P <0.001 in two-sample *t *test between control BMMSCs and infected BMMSCs. ΔΨ_m_, mitochondrial inner membrane potential; CCCP, carbonyl cyanide m-chlorophenylhydrazone; JC-10, enhanced 5,5',6,6'-tetrachloro-1,1',3,3'-tetraethylbenzimidazolcarbocyanine iodide.

BMMSC lines 407, 470, and 412 were used to study the early effects of pathogen contamination on the state of mitochondrial energy of BMMSCs. Based on the above results with Trypan blue staining and analysis of cell morphology, which showed that changes begin to appear in the viability of BMMSCs after 6 hours of infection, the 4-hour time point was chosen for ΔΨ_m _analysis. The aim was to investigate whether changes in mitochondrial energy state precede the changes that can be detected by traditional Trypan blue staining. In these experiments, CCCP treatment was used as a positive control to depolarize the ΔΨ_m _(Figure 3a). It was evident that the *S. aureus *strains had a slightly different effect on the cells. Namely, with *S. aureus *Cowan I only, the BMMSC line 412 showed a significant decrease in ΔΨ_m _from 100% ± 5.8% to 84.9% ± 6.6% when the highest inoculum of *S. aureus *Cowan I was used and the ΔΨ_m _of infected BMMSCs was compared with the ΔΨ_m _of control BMMSCs (*P *<0.05) (Figure 3b). Instead, *S. aureus *KK1089 induced a significant decrease in ΔΨ_m _under most experimental conditions in all three BMMSC lines even before any major changes in viability or morphology were evident (Figure 3c). The initial inoculum of 10^3 ^of *S. aureus *KK1089 caused significant depolarization of ΔΨ_m _in all BMMSC lines from 100% ± 5.8% to 56.8% ± 7.4% (*P *<0.01) in BMMSC 407, from 100% ± 5.8% to 77.7% ± 2.6% (*P *<0.001) in BMMSC 412, and from 100% ± 5.8% to 85.3% ± 4.3% (*P *<0.01) in BMMSC 470. *S. epidermidis *ATCC 14990 and *S. epidermidis *KK1087 also induced significant depolarization of ΔΨ_m _in all three BMMSC lines at all microbial loads when compared with uninfected control cells (Figure 3d, e). The initial bacterial load of 10^3 ^of *S. epidermidis *KK1087 caused dramatic decreases of ΔΨ_m _in 407, 412, and 470 BMMSC lines from 100% ± 5.8% to 58.1% ± 20.1% (*P *<0.05), from 100% ± 5.8% to 73.6% ± 8.6% (*P *<0.01), and from 100% ± 5.8% to 81.1% ± 9.6% (*P *<0.05), respectively. *P. aeruginosa *PA01 caused depolarization of ΔΨ_m _in all BMMSC lines at higher bacterial loads. The ΔΨ_m _decreased from 100% ± 5.8% to 65.4% ± 11.9% in BMMSC 407 line (*P *<0.01), from 100% ± 5.8% to 89.1% ± 5.2% in BMMSC 412 line (*P *<0.05), and from 100% ± 5.8% to 90.0% ± 3.1% in BMMSC 470 line (*P *<0.01). However, the lowest bacterial loads did not induce a significant decrease of ΔΨ_m _in the BMMSC line 407 when compared with control cells (Figure 3f). The results clearly indicate that the depolarization of ΔΨ_m _occurred before major changes in morphology or in viability as determined by Trypan blue staining were evident, except with the highest amount of *P. aeruginosa *PA01 (Figure 3f). Trypan blue staining results are shown for the BMMSC line 412 only, but the results were identical for the two other BMMSC lines. The results also show that the level of depolarization differed among the three BMMSC lines. Overall, cell line 407 showed the most dramatic depolarization in response to all bacterial strains which caused significant depolarization of ΔΨ_m _(all except *S. aureus *Cowan I), and this line also showed the most dramatic response to CCCP. The BMMSC line 412 showed a somewhat different response to CCCP when compared with the two other BMMSC lines.

### All bacterial strains grew exponentially within 24 hours of infection, but there were some variations in bacterial growth during the first 4 hours of infection

**Figure 4:**
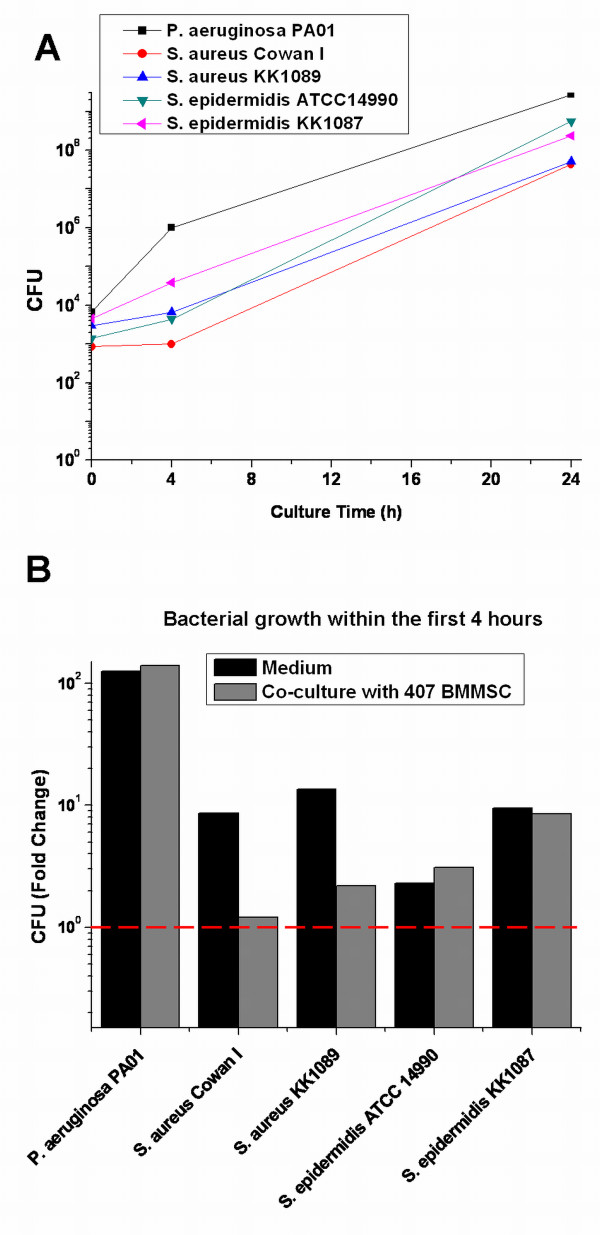
**Determination of bacterial growth in co-culture with bone marrow-derived mesenchymal stem cells (BMMSCs)**. BMMSC line 407 was used to determine the bacterial growth rate in co-culture system. Identical culture conditions without BMMSCs were used as a control. *Pseudomonas aeruginosa *PA01, *Staphylococcus aureus *strains Cowan I and KK1089, and *Staphylococcus epidermidis *strains ATCC14990 and KK1087 were inoculated at an indicated initial inoculum (0 hours), and bacterial growth was determined by viable counting after 4 and 24 hours of infection. Series of 10-fold dilutions were prepared from each sample, and the number of colony-forming units (CFU) was determined from at least two different 10-fold dilutions per sample. **(a) **All the bacterial strains grew exponentially within 24 hours of infection. However, there were some differences in growth rate at the beginning of the infection. **(b) ***P. aeruginosa *PA01 showed rapid proliferation whereas *S. aureus *Cowan I showed negligent growth in the presence of BMMSCs within the first 4 hours of infection. Moreover, both *S. aureus *strains grew more rapidly in medium without BMMSCs within the first 4 hours than when compared with bacterial growth on top of the BMMSCs. Both *S. epidermidis *strains showed relatively active proliferation within the first 4 hours of infection.

Next, we determined bacterial growth in co-culture with BMMSCs after 4- and 24-hour infection. The initial inoculum of approximately 10^3 ^bacteria was seeded onto the 407 BMMSCs. As BMMSCs have been reported to have antimicrobial effects [36,37], we inoculated bacteria for comparison into cell culture medium not containing BMMSCs. Quantitative counting of bacteria showed that there was some variation in bacterial numbers in the initial inocula; however, the amounts of all strains were in the order of magnitude of 10^3^. All bacterial strains showed exponential growth within 24 hours of infection when grown on top of the BMMSCs (Figure 4a). At 24 hours, efficient growth was also detected when bacteria were incubated in plain medium (data not shown). However, within the first 4 hours of infection, there were significant differences in growth between the different strains (Figure 4b). The amount of *P. aeruginosa *PA01 increased 139 times during the first 4 hours of infection when grown on top of the BMMSCs and 125 times in the medium only. On the other hand, *S. aureus *Cowan I showed negligent growth during the first 4 hours of infection on top of the BMMSCs whereas in the plain medium the amount of *S. aureus *Cowan I increased nine times. *S. aureus *KK1089 behaved similarly but grew to consistently higher numbers than *S. aureus *Cowan I both in the plain medium (14-fold increase) and on top of the BMMSCs (over twofold increase). *S. epidermidis *ATCC14990 showed over threefold increase in bacterial amounts when grown on top of the BMMSCs and over twofold increase in the medium only. Also, *S. epidermidis *KK1087 showed rapid proliferation within the first 4 hours of infection with CFU fold changes of nine in co-culture with BMMSCs and nine in medium only. The results show that *S. aureus *Cowan I in particular proliferates slowly during the first 4 hours of infection. This may explain the inability of the mitochondrial assay to detect *S. aureus *Cowan I-induced cellular stress within the first 4 hours of infection. We also determined bacterial proliferation with the initial inocula of approximately 10^1 ^and 10^2 ^bacteria per well and noted that the bacterial growth curves were similar to those obtained with the highest inoculum (data not shown). The results also show that growth of the *S. aureus *strains is significantly slower in the presence of BMMSCs than in plain medium, suggesting that antimicrobial effects from the cells may hinder their growth.

### Mitochondrial assay showed sensitivity for reference strains used in microbial quality control of cellular products

**Figure 5:**
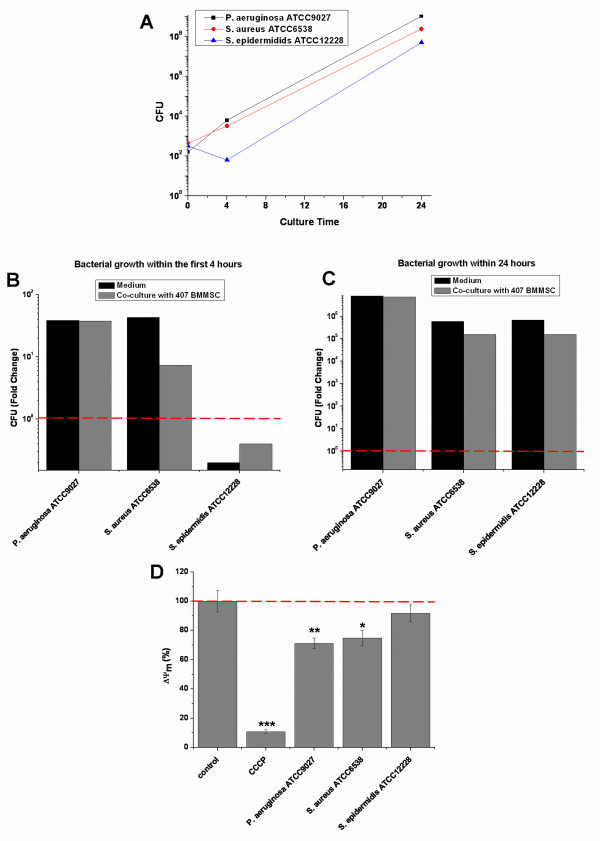
**Comparison of ΔΨ_m _of bone marrow-derived mesenchymal stem cells (BMMSCs) and bacterial viability in co-culture of BMMSCs with reference strains *Pseudomonas aeruginosa *ATCC9027, *Staphylococcus aureus *ATCC6528, and *Staphylococcus epidermidis *ATCC12228**. BMMSC line 407 was used to determine the bacterial growth kinetics within 4 and 24 hours of infection. *P. aeruginosa *ATCC9027, *S. aureus *ATCC6538, and *S. epidermidis *ATCC12228 were inoculated in an indicated initial inoculum (0 hours). Medium without BMMSCs was used as a control. Series of 10-fold dilutions were prepared from each sample, and the number of colony-forming units (CFU) was determined from at least two different 10-fold dilutions per sample. **(a) **All of the bacterial strains grew exponentially within 24 hours of infection. **(b) **There were some differences in the growth rate of bacteria between different bacterial strains within the first 4 hours of infection. **(c) **At this time point, initially slowly proliferated *S. epidermidis *ATCC12228 also showed efficient bacterial growth. **(d) **The analysis of ΔΨ_m _was performed in parallel with the determination of CFUs after 4 hours of infection. The fluorescent 595 nm/535 nm ratio of JC-10 was used to determine ΔΨ_m_, and the 595 nm/535 nm ratios of infected BMMSCs were compared with controls. Results are indicated as mean ± standard deviation of four independent replicates. *Two-sample *t *test *P *values of less than 0.05 when compared between control BMMSCs and infected BMMSCs; **two-sample *t *test *P *values of less than 0.01 when compared between control BMMSCs and infected BMMSCs; ***two-sample *t *test *P *values of less than 0.001 when compared between control BMMSCs and CCCP-treated BMMSCs. ΔΨ_m_, mitochondrial inner membrane potential; CCCP, carbonyl cyanide m-chlorophenylhydrazone; JC-10, enhanced 5,5',6,6'-tetrachloro-1,1',3,3'-tetraethylbenzimidazolcarbocyanine iodide.

Finally, we studied the potential of ΔΨ_m _measurement to detect bacterium infection-induced changes in viability of BMMSCs by using certified reference microbial strains that are widely used in validation of quality control processes of cell therapy products. We measured ΔΨ_m _and bacterial proliferation after infection of BMMSC 407 with the strains *P. aeruginosa *ATCC9027, *S. aureus *ATCC6538, and *S. epidermidis *ATCC12228. Again, the bacteria were seeded onto the 407 BMMSCs as well as into medium not containing BMMSCs, and bacterial growth was detected by counting CFU after 4 and 24 hours of infection. All bacterial strains grew exponentially within 24 hours of infection (Figure 5a). However, with reference strains, there were some variations in the proliferation within the first 4 hours of infection (Figure 5a, b). Interestingly, similar to *S. aureus *Cowan I and *S. aureus *KK1087, *S. aureus *ATCC6538 grew to significantly higher numbers in the absence of BMMSCs (fold change of 43) than in the presence of cells (fold change of 7), again suggesting that BMMSCs may have antimicrobial effects toward *S. aureus *(Figure 5b). *P. aeruginosa *ATCC9027 showed the highest growth with a fold change of close to 40 in both culture conditions, whereas the number of *S. epidermidis *ATCC12228 even declined within the first 4 hours of infection. On the other hand, even though there were differences in bacterial growth at the beginning, all bacterial strains, including *S. epidermidis *ATCC12228, grew efficiently during 24 hours of infection (Figure 5c). *S. aureus *ATCC6538 continued to show higher proliferation in medium only (fold change of 6 × 10^5^) when compared with the growth in co-culture with BMMSCs (fold change of 2 × 10^5^) also during prolonged incubation. Similarly, *S. epidermidis *ATCC12228 proliferated more in medium only (fold change of 7 × 10^5^) than when cultivated on top of the BMMSCs (fold change of 2 × 10^5^) (Figure 5c).

In parallel with the determination of bacterial CFU, we performed the analysis of ΔΨ_m _within 4 hours of infection. Results are represented as a mean ± SD of four independent replicates (Figure 5d). Again, CCCP was used as a positive control, and it induced total depolarization of ΔΨ_m _from 100% ± 7.2% to 10.8% ± 1.3% (*P *<0.001) when compared with control BMMSCs. *P. aeruginosa *ATCC9027, which showed rapid proliferation within the first 4 hours of infection, also induced significant depolarization of ΔΨ_m _of BMMSCs from 100% ± 7.2% to 71.2% ± 3.7% (*P *<0.01). In addition, *S. aureus *ATCC6538 induced the depolarization of ΔΨ_m _of BMMSCs from 100% ± 7.2% to 74.9% ± 4.9% (*P *<0.05) when compared with control BMMSCs. On the contrary, *S. epidermidis *ATCC12228 caused only a slight depolarization of ΔΨ_m _of BMMSCs with the change from 100% ± 7.2% to 91.9% ± 5.6%. Reference strains behaved somewhat similarly as the strains used in earlier experiments: after 24 hours of infection, total dissipation of BMMSCs was evident with *P. aeruginosa *ATCC9027 and *S. epidermidis *ATCC12228 whereas *S. aureus *ATCC6538 formed enormous aggregates (data not shown). Moreover, after 6 hours of infection, *P. aeruginosa *ATCC9027-induced morphological changes in BMMSCs and *S. aureus *ATCC6538 formed aggregates as did the two other *S. aureus *strains whereas *S. epidermidis *ATCC12228 did not induce any visible changes in BMMSCs (data not shown). These results indicate that the results from the mitochondrial assay were well in line with bacterial proliferation within the first 4 hours of infection. All together, the results with the reference strains support the results obtained with other bacterial strains.

## Discussion

The risk of microbial contamination is well recognized in the propagation of cells for therapeutic use, but little has been published to demonstrate and characterize possible effects of contamination and infection on stem cell properties and hence on the viability and ultimately on the safety monitoring of cell therapy products [10,12,14-16]. Accordingly, in the present study, we chose to focus on the quality control point of view of microbial risks during the *in vitro *manufacture of BMMSC-based cellular products. The present study shows that infection of BMMSCs with even low numbers of bacteria inflicts serious damage on BMMSC cultures. *P. aeruginosa *PA01, *P. aeruginosa *ATCC9027, and all *S*. *epidermidis *strains caused total dissipation of the BMMSC cultures within 24 hours of infection. *S. aureus *KK1089, *S. aureus *ATCC 6538, and *S. aureus *Cowan I also caused a decrease in the viability of BMMSCs but behaved somewhat differently by forming aggregates on the top of BMMSC monolayers. The exact content of such aggregates remains unknown at this time but is likely to be due to the coagulase protein of *S. aureus *which reacts with prothrombin in blood and enables conversion of fibrinogen to fibrin, leading to blood clotting [38]. Remnants of coagulation cascade proteins in FBS may react with *S. aureus *coagulase and cause aggregation of bacteria. As opposed to *S. aureus*, *S. epidermidis *strains are coagulase-negative, and this probably explains the absence of aggregates in samples infected with *S. epidermidis*.

Even though *S. epidermidis *generally may be regarded as less virulent than *S. aureus *and *P. aeruginosa *[20], all three *S. epidermidis *strains in our study showed definite deleterious effects on BMMSCs, as they also decreased the ΔΨ_m _and caused dissipation of BMMSCs. The results thus suggest that an opportunistic pathogen, such as *S. epidermidis*, is equally hazardous to stem cells as the more virulent pathogens. Overall, during prolonged incubation, all pathogens in the present study promoted somewhat similar levels of cell destruction regardless of the amount of the initial inoculum.

Quantitative counting of bacteria showed that *P. aeruginosa *strains proliferated actively from the beginning but that other strains showed lower growth. In particular, *S. aureus *Cowan I showed almost negligent bacterial growth within the first 4 hours of infection and seemed to need more time to adapt to the BMMSC culture medium. Subsequently, *S. aureus *Cowan I also began to cause a significant decrease in stem cell viability. The damage to BMMSCs was evident after 6 hours of infection as demonstrated by traditional live/dead assay, Trypan blue staining, and monitoring of cellular morphology. As expected, *P. aeruginosa *PA01 caused a clear decrease in viability of BMMSCs after 6 hours of infection and this is well in line with the rapid bacterial growth within the first 4 hours of infection. Also, other strains caused an increase in the number of Trypan blue-stained nuclei of BMMSCs within the first 6 hours of infection, even though the cellular damage was not as apparent as with *P. aeruginosa *strains. Accordingly, it seems that the signs of pathogen-induced cellular damage can be seen after 6 hours of infection with traditional viability methods. The result clearly demonstrates the risk associated with microbial contamination and infection. Therefore, methods for validation and quality control of cell and tissue products call for systematic monitoring of microbial contamination but also for reliable methods for monitoring the viability of the cell product and the possible deleterious effects of infection of stem cells during manufacturing.

Interestingly, the BMMSC line 407 appeared to show antimicrobial activity toward all three *S. aureus *strains studied. Proliferation of *S. aureus *strains was lower when grown on top of BMMSCs than in the medium without the cells. Antimicrobial activity of BMMSCs has recently been reported in two studies [36,37]. Krasnodembskaya and colleagues [36] (2010) showed that hMSCs possess antimicrobial activity toward *Escherichia coli *K1, *P. aeruginosa *strain PA103, and *S. aureus *strain Newman. Moreover, they showed that intratracheal administration of hMSCs decreased bacterial growth in an *in vivo *mouse model of *E. coli *pneumonia. This was shown to be mediated by the secretion of the antimicrobial peptide LL-37 [36]. Meisel and colleagues [37] (2011) postulated that hMSCs show antimicrobial activity toward *S. aureus *and *P. aeruginosa *and that the activity is enhanced when hMSCs are pretreated with interferon gamma (IFN-γ). It was also shown that the antimicrobial function is mediated by indoleamine 2,3-dioxygenase (IDO) activity, which also plays a major role in immunosuppression activity of hMSCs, and is induced by IFN-γ [39]. In our assay conditions, which did not include IFN-γ, no significant effects toward *P. aeruginosa *were noted. However, our results with *S. aureus *support earlier findings on the antimicrobial activity of MSCs.

Mitochondria are well-known targets of many bacterial toxins and compounds [40,41]. It has been demonstrated that *S. aureus *directly targets host mitochondria and induces apoptosis through α-toxins and Panton-Valentine leukocidin [42,43]. In addition, *P. aeruginosa *and *S. epidermidis *have been shown to induce mitochondria-mediated apoptosis in infected host cells [44,45]. A relevant question, therefore, is whether changes in ΔΨ_m _are already present before plasma membrane breakout and morphological changes are detected. In the present study, we tested the suitability of mitochondrial analyses as a quality control tool for BMMSCs to detect microbial infection-induced cellular stress. To perform this experiment in accordance with current microbial quality control methods, we also used reference strains which are used in routine quality control studies by cell and tissue banks. All pathogens except *S. aureus *Cowan I and *S. epidermidis *ATCC12228 caused systematic depolarization of ΔΨ_m _after only 4 hours of infection. However, at that time point, only the highest loads of *P. aeruginosa *PA01 had induced visible changes in cell membrane integrity, whereas no major changes were evident with the other bacteria. The inability of the mitochondrial assay to detect the impact of *S. aureus *Cowan I and *S. epidermidis *ATCC12228 on the ΔΨ_m _of BMMSCs may be explained by their negligent growth or even a decline in bacterial number within the first 4 hours of infection. Therefore, the results from bacterial growth analysis are well in line with the results from the mitochondrial assay, confirming the sensitivity of the method. The reason for the negligent or decreased bacterial growth remains unknown, but it may be that these strains need more time for adapting to BMMSC culture conditions since they both did show exponential growth during a longer term of infection. Moreover, antimicrobial effects hindering the proliferation of *S. aureus *Cowan I in co-culture with BMMSCs may partly explain the slow growth of *S. aureus *Cowan I. In some cases, even the lowest numbers of bacteria caused a significant decrease in ΔΨ_m _when compared with uninfected control stem cells. Thus, measurement of ΔΨ_m _offers a sensitive method for detection of microbial damage to cell products. Interestingly, the three BMMSC lines showed different levels of depolarization of ΔΨ_m _after bacterial infection. The reason for such a difference remains speculative at this point and warrants further studies. However, we previously demonstrated that the level of ΔΨ_m _corresponds to the function and differentiation potency of BMMSCs [33]. Therefore, the development of rapid diagnostic tests based on mitochondrial function is urgently called for to ensure the viability and potency of stem cell products.

## Conclusions

To conclude, co-culture of BMMSCs in the presence of *S. aureus*, *S. epidermidis*, and *P. aeruginosa *damaged the viability of BMMSCs as measured by ΔΨ_m_. Such deleterious effects raise significant concerns for the safety and efficacy of stem cell products for use in clinical therapy. Additionally, a relatively short exposure to antibiotic-free medium can reveal a concealed and suppressed infection. Even low levels of bacterial contamination can be a major hazard for patients as such, and the deleterious effects of even short exposure to bacterial pathogens may seriously alter the efficacy of cellular therapy. On the other hand, by monitoring of the energy state of stem cell mitochondria, it is possible to detect changes in infected host cells even before any other changes are evident by Trypan blue staining or microscopy for cell morphology. Accordingly, mitochondrial analyses show strong potential for quality control and monitoring of safety and efficacy of cell-based products. In addition, in compliance with regulatory demands for microbial safety, techniques to detect low levels of bacterial contamination in a matter of hours are urgently called for and should be convenient to incorporate into testing for mitochondrial function.

## Abbreviations

αMEM: alpha minimum essential medium; ΔΨ_m_: mitochondrial inner membrane potential; BMMSC: human bone marrow-derived mesenchymal stem cell; CCCP: carbonyl cyanide m-chlorophenylhydrazone; CFU: colony-forming unit; FBS: fetal bovine serum; hMSC: human mesenchymal stem cell; IDO: indoleamine 2,3-dioxygenase; IFN-γ: interferon gamma; JC-10: enhanced 5,5',6,6'-tetrachloro-1,1',3,3'-tetraethylbenzimidazolcarbocyanine iodide; PBS: phosphate-buffered saline; SD: standard deviation; TSA: trypticase soy agar.

## Competing interests

The authors declare that they have no competing interests.

## Authors' contributions

MP carried out the cell and microbial culture, microbial infection of BMMSCs, and analysis of cellular viability; contributed to the study design; and wrote the manuscript. KL contributed to the study design and coordination, helped in experiments, participated in analysis of results, and helped to draft the manuscript. SL contributed to the study design, helped to draft the manuscript, established the BMMSC culture protocol, and helped in experiments. H-VL provided BMMSC material, contributed to the study design, and helped to draft the manuscript. MN contributed to the study design, helped to draft the manuscript according to microbial part, and contributed to the data analysis. ML isolated microbial material and contributed to the study design. JM contributed to the study design and coordination and helped to draft the manuscript. PL contributed to the study design and coordination, helped to draft the manuscript, and contributed to the data analysis. KN participated in study design and coordination and helped to draft the manuscript. All authors have read and approved the final manuscript.
